# Supplementary material for: Transcriptional Reprogramming of Candida tropicalis in Response to Isoespintanol Treatment
Source: J Fungi (Basel). 2023 Dec 15;9(12):1199. doi: 10.3390/jof9121199 (PMC10744401; doi:10.3390/jof9121199)
Supplement: Supplementary file 1 [file jof-09-01199-s001.zip › jof-2738935-supplementary.pdf]

Journal of Fungi

MDPI

Article

Transcriptional Reprogramming of Candida tropicalis in Response to Isoespintanol Treatment

Orfa Inés Contreras Martínez[1]*, Alberto Angulo Ortíz[2], Gilmar Santafé Patiño[2], Katia Aviña-Padilla[3] María Camila Velasco Pareja[4] and María Fernanda Yasnot [4]

[1] Biology Department, Faculty of Basic Sciences, University of Córdoba, Montería 230002, Colombia
[2] Chemistry Department, Faculty of Basic Sciences, University of Córdoba, Montería 230002, Colombia
[3] Center for Research and Advanced Studies of the I.P.N Unit Irapuato, Irapuato 36821, Mexico
[4] Bacteriology Department, Faculty of Health Sciences, University of Córdoba, Montería 230002, Colombia
* Correspondence: oicontreras@correo.unicordoba.edu.co

Supplementary Materials

Table S1. List of real-time qPCR primers

Genes	Sequences
ERG6	fwd: 5′-CAG.TCT.TGG.AAG.GTG.TCT.ACT.C-3′
	rev: 5′- GGT.GTT.CTT.CGT.TGG.TTT.CAT.C-3′
KRE1	fwd: 5′-GCA.GAT.GAA.ACC.ACA.TCA.TCA.TC-3′
	rev: 5′-CAT.CAG.TAC.CTG.TTA.CCC.AAA.CTA-3′
CTRG_03786	fwd: 5′- GGT.GGT.ACT.GAT.ACC.GTT.GTT -3′
	rev: 5′- GTG.GGT.TGG.TGT.AGG.TGA.TAG -3′
ACTIN-1	fwd: 5′-GAC.CGA.AGC.TCC.AAT.GAA.TC-3′
	rev: 5′-AAT.TGG.GAC.AAC.GTG.GGT.AA-3′

Table S2. RNA extraction of *C. tropicalis*. Quality control total RNA extracted from Zoea-type larvae of *Callinectus sapidus*

Sample Code	Purified RNA [ng/uL]	Final volume (uL)	Total purified RNA [ug]
RNAISO	29,51	27	0,797
RNAINO	32,701	24	0,785

RNAISO: ISO treated Yeast; RNAINO: untreated yeast used as control

Table S3. NGS sequencing by RNA-seq of *C. tropicalis*

Sample	Total reads	Clean reads %
RNAISO	40451606	94.9
RNAINO	26484468	97.6

Table S4. Mapping of transcriptomes obtained by RNA-seq

	RNAISO	RNAINO
Number of input reads	19832040	13126552
Average input read length	292	297
Uniquely mapped reads number	16219548	11109083
Uniquely mapped reads %	81.78%	84.63%
Average mapped length	288.70	294.30
